# 

**Published:** 1899-05

**Authors:** 


THE BRAZOS VALLEY MEDICAL ASSOCIATION will hold its Seventh Semi-Annual Meeting in Marlin, Texas, May 9 and 10, inst. Dr. G. R. Tabor, of Bryan, President, Dr. W. B. Briggs, Easterly, Secretary. A most interesting program has been prepared.

